# Regulators of Rho GTPases in the Nervous System: Molecular Implication in Axon Guidance and Neurological Disorders

**DOI:** 10.3390/ijms20061497

**Published:** 2019-03-25

**Authors:** Sadig Niftullayev, Nathalie Lamarche-Vane

**Affiliations:** 1 Cancer Research Program, Research Institute of the MUHC, Montreal, QC H4A 3J1, Canada; sadig.niftullayev@mail.mcgill.ca; 2 Department of Anatomy and Cell Biology, McGill University, Montreal, QC H3A 2B2, Canada

**Keywords:** Rho GTPases, GAPs, GEFs, axon guidance, neurological diseases, central nervous system

## Abstract

One of the fundamental steps during development of the nervous system is the formation of proper connections between neurons and their target cells—a process called neural wiring, failure of which causes neurological disorders ranging from autism to Down’s syndrome. Axons navigate through the complex environment of a developing embryo toward their targets, which can be far away from their cell bodies. Successful implementation of neuronal wiring, which is crucial for fulfillment of all behavioral functions, is achieved through an intimate interplay between axon guidance and neural activity. In this review, our focus will be on axon pathfinding and the implication of some of its downstream molecular components in neurological disorders. More precisely, we will talk about axon guidance and the molecules implicated in this process. After, we will briefly review the Rho family of small GTPases, their regulators, and their involvement in downstream signaling pathways of the axon guidance cues/receptor complexes. We will then proceed to the final and main part of this review, where we will thoroughly comment on the implication of the regulators for Rho GTPases—GEFs (Guanine nucleotide Exchange Factors) and GAPs (GTPase-activating Proteins)—in neurological diseases and disorders.

## 1. Axon Guidance

**Figure 1:**
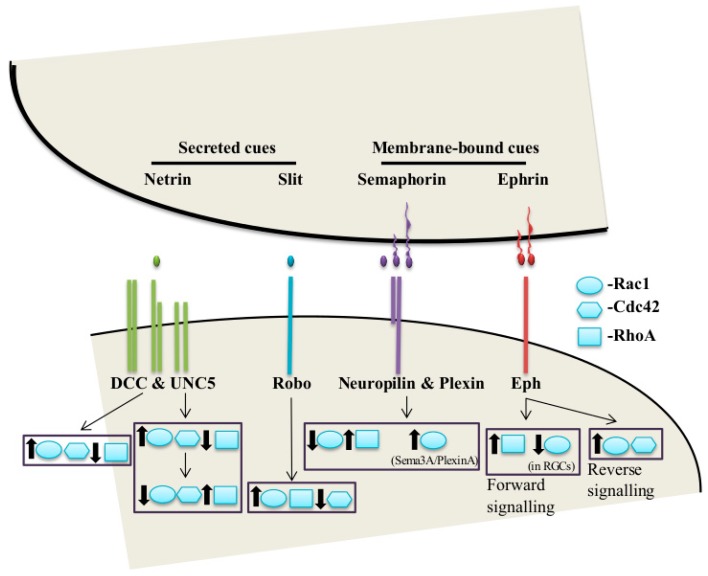
The classical guidance cues, their receptors, and their effect on Rho GTPases. A schematic representation of the up- or downregulation (bold arrows) of RhoA, Rac1, and Cdc42 by different guidance cue/receptor combinations.

Species with bilateral symmetry, including humans, possess the midline axis, which is a very important structure for proper wiring of the nervous system [1,2,3,4,5,6]. This feature becomes even more crucial in the vertebrates because of the complexness of the nervous system where neurons have to decide whether or not to cross the midline or towards which axis (for example, rostral versus caudal or ventral versus dorsal) they should send their axons to [7]. Currently, it is known that the axons of later-born neurons fasciculate with the axons of early-born neurons or simply follow the scaffold that had been laid by them. The early born neurons, however, have to navigate through an environment that is composed of undifferentiated neuroepithelial cells [8]. Pioneering studies addressing this issue dates back to late 19th century, to the work of the “father of neuroscience”—Ramon y Cajal. More than a century ago, he observed that the distal tip of the axon has a very irregularly shaped structure, which he called “the growth cone” [5,9,10]. Cajal proposed that the growth cone might be the structure that, in response to diffusible chemotropic signals, navigates through the developing brain to connect with the distant targets [9,11]. Approximately two decades later, motility of the growth cone was shown by Harrison, using frog neurons [12,13,14]. The theory became more substantiated by the work of Sperry, where he showed that specific populations of retinal axons innervate only certain parts of the optic tectum, suggesting that there is some sort of molecular complementation between these axons and the tectum [15]. The influx of data supporting the possible chemoattraction model encouraged the researchers to reveal the underlying molecular components. Many of the early candidates, such as neural cell adhesion molecules (NCAMs), integrins, fasciculin, and cadherins, did not fit the model since they were simply providing permissive environment rather than conferring directionality to the navigating axons [5]. Finally, a little less than a century after Cajal’s initial work, using the combination of genetic, biochemical, in vitro, and in vivo approaches, scientists identified the classical guidance cues—Netrins, Slits, Semaphorins, and Ephrins—and their cognate receptors—deleted in colorectal cancer (DCC) and uncoordinated-5 (UNC5), Roundabout (Robo), Plexin and Neuropilin, and Eph, respectively [5,16,17] (Figure 1). As a result of the work done during the past few decades, it is now known that the growth cone is a very motile structure, mainly composed of highly dynamic cytoskeletal elements—actin and tubulin filaments—as well as, a myriad of other proteins, which participate in the regulation of cytoskeletal assembly/disassembly. The past research has also disclosed the fact that the growth cone is furnished with one or more of the above-mentioned receptors to be able to respond to the guidance cues appropriately and to be able to navigate accordingly [3,18,19,20].

The importance of the axon guidance cues and their receptors can also be seen from the fact that several studies have demonstrated a link between single nucleotide polymorphisms (SNPs) and mutations in the genes encoding these proteins and numerous congenital disorders and neurodegenerative diseases such as Parkinson’s disease (PD), Alzheimer’s disease (AD), Mirror movement disorder (MMD), dyslexia, Kallmann’s syndrome, Hirschsprung’s disease, Autism spectrum disorders (ASD), epilepsy, and Amyotrophic lateral sclerosis (ALS) [17,20,21].

Although some of the well-known morphogens such as Wnt, Sonic hedgehog (Shh), and transforming growth factor β (TGFβ)/bone morphogenetic protein (BMP) families have also been implicated in axon guidance [3,4,16,17], we will only discuss the classical guidance cues that have been mentioned above. Also, it must be noted that the classical guidance cues are not restricted to the central nervous system (CNS), but also function in various molecular pathways outside the CNS [22,23], however, this will not be discussed here.

## 2. The Classical Guidance Cues and Their Receptors in Axon Guidance

### 2.1. Netrins

The netrins are members of a family of conserved secreted guidance cues. Netrins were uncovered in a convergent series of experiments that were carried out on invertebrates (circumferential axon guidance in *C. elegans*) and vertebrates (ventromedial guidance of commissural axons in vertebrates) trying to identify the molecules that function as chemoattractants in axon guidance [5,16,17]. The discovery of netrins in different branches of the animal kingdom proved that, despite the increasing complexity of the nervous system through evolution, some of the underling mechanisms have been well conserved [17]. So far, one netrin has been identified in *C. elegans* (UNC-6), two in *Drosophila* (netrin-A and B), and several in vertebrates—secreted netrins, netrin-1 to -5, and glycosylphosphatidylinositol (GPI)-anchored membrane-bound netrins—netrin-G1 and -G2. Mammals, including rats, mice, and humans, express netrin-1, -3, -4, -5, -G1, and -G2. Netrin-2 expression, however, has only been detected in chicken and zebrafish [24,25]. The N-terminal domain of netrins is homologous to the domains V and VI found at the amino terminal ends of laminins. In that sense, netrin-1, -2, and -3 show similarity to γ chain of laminins, while netrin-4 and netrin-Gs are similar to the β chain of laminins [16,24].

Further research has shown that, in vertebrates, netrin-1 is produced by the floor plate and functions as a gradient to attract commissural axons [5,16,17]. This classical model has been challenged by a number of recent studies demonstrating that the ventricular zone-derived netrin-1, but not the floor plate, is required for hindbrain commissural axon attraction to the ventral midline [26,27,28]. However, a recent study suggested a synergistic role of the floor plate and ventricular zone-derived netrin-1 in commissural axon guidance [29]. Additionally, netrin-1 can also act as a chemorepulsive cue, repelling trochlear motor neurons and other dorsally projecting hindbrain motor neurons [5]. The chemoattractive functions of netrin-1 are mediated by “deleted in colorectal cancer” (DCC) family of proteins (UNC-40 in *C. elegans*, Frazzled in *Drosophila*), characterized by four immunoglobulin (Ig) and six fibronectin (Fn) type III repeats in their extracellular domains [5,16,17,24]. Netrin-1 has also been shown to bind to another DCC family member, neogenin, and the Ig superfamily member, Down syndrome cell adhesion molecule (DsCAM), in other systems [16]. On the other hand, the repulsive functions of netrin-1 are mediated by the proteins of the UNC family, of which there is one (UNC5) in *C. elegans and Drosophila* and four in mammals (UNC5A-D). In *Drosophila*, UNC5 carries out the repulsive response of netrin alone, while in *Xenopus,* UNC5 and DCC form heterodimers to mediate the repulsion [30]. In *C. elegans*, however, there are multiple repulsive signaling mechanisms involving UNC5 and UNC-40 [31].

### 2.2. Slits

Slits are large secreted chemorepellents. They contain four N-terminal leucine-rich repeats, as well as, epidermal growth factor (EGF)-like repeats [16]. Much of our understanding of slits are coming from work carried out in *Drosophila* where they were discovered as ligands of the receptor protein roundabout (robo), which ensures that neurons cannot re-cross the midline, after having crossed it once [5,16,17]. There has been only one identified slit protein in *C. elegans* and *Drosophila*, versus three slits in mammals (Slit1-3). In parallel studies on *C. elegans*, robo was identified as a regulator of nerve ring formation [17]. There are three Robos in *Drosophila*, three robos in mammals, and only one (Sax-3) in *C. elegans* [16]. Slit-robo pathway was another evidence for the conservation of the axon guidance mechanisms through evolution [17].

In vertebrates, slit has been shown to control the midline crossing of the axons, working along with netrins. However, it was only after elimination of all three slit isoforms that its importance could be finally shown in term of commissure formation [17]. Furthermore, the expression of different robo combinations controls the degree of responsiveness to slit and consequently, determines the exact position of the neurons, once they cross the midline [5,16,17].

The expression of both Robo and DCC in commissural axons begs the question of “How do these axons cross the midline?” or “How do these axons cross the midline only once?”. In *Drosophila*, another protein, namely commissureless (comm), inhibits surface expression of Robo, by targeting it to proteasomal degradation, in the precrossing axons. Once the axon crosses the midline, the expression level of comm decreases, leading to an increase in the surface expression of robo, which prevents recrossing of axons. Even though, there has not been any identified comm homolog in mammals, it has been noted that robo3 produces two isoforms by alternative splicing: robo3.1, which is highly expressed in the precrossing axons and inhibits the repulsive action of robo1 and 2; and robo3.2, which is highly expressed in the postcrossing axons and enhances the repulsive action of the robo1 and 2 [5,17]. Furthermore, it has been shown that upregulation of robo levels after midline crossing act in two ways: (1) by increasing the slit responsiveness (2) and by directly interacting with the cytoplasmic tail of DCC (in *cis* manner) to attenuate netrin-1-DCC signaling [5].

In addition to its role in midline crossing of axons, Slit-Robo signaling is also involved in the repulsion of retinal axons at the optic chiasm, in the repulsion of neuronal precursors migrating to the olfactory bulb, and in the repulsion of olfactory bulb axons. Another proven role of slit in the vertebrate nervous system is its role as a branching factor for sensory axons and cortical dendrites [17].

### 2.3. Semaphorins

Semaphorins (sema) represent a large family of phylogenetically conserved guidance cues that include both secreted and membrane-bound proteins, all of which contain the semaphorin domain of 500 amino acids that mediate receptor interaction. The first identified semaphorin—Fasciclin IV (Sema-1A)—is a transmembrane protein that is required for pathfinding in grasshopper limb sensory neurons [16]. The first identified vertebrate semaphorin, on the other hand, was a secreted protein—Collapsin-1 (Sema-3A), which was discovered from chick brain extracts because of its ability to cause growth cone collapse in cultured neurons [5,16,17]. Today, around twenty members of semaphorins have been discovered and divided into eight classes, based on their structural homology—class 1 and 2 are invertebrate semaphorins, whereas classes 3–7 are from vertebrates. However, emerging evidence make this lineation more and more ambiguous [17].

The first identified semaphorin receptor was neuropilin (Npn), of which there are only two members in vertebrates, neuropilin-1 and -2, and none in invertebrates. However, the lack of any signaling domains in neuropilins ignited the search for an alternative receptor. This led to the discovery of Plexins, a phylogenetically conserved family that is distantly related to the Semaphorins, which contains more than ten members under four different subfamilies (PlexinA–D). Different permutations of plexin and neuropilin receptors are expressed in distinct subset of neurons [17].

In vertebrates, the role of semaphorins in axon guidance has been studied mostly through secreted class-3 Semaphorins [17]. For example, Sema3A has been shown to be expressed in tissues that surround peripheral nerves, where it repels axons from entering into wrong trajectories by a mechanism called “surround repulsion” [16]. Generally, defects in distinct members of Sema3 signaling has been shown to cause aberrant projections of sensory neurons and specific motor cranial nerves (oculomotor, trochlear, trigeminal, and facial nerves), as well as defasciculation of nerve bundles [5]. The transmembrane semaphorins act also as repellents either through surround repulsion or by direct expression on the nerve bundles. In addition to their well-known repulsive roles, semaphorins have also been implicated in chemoattraction for cortical dendrites [17]. Another interesting aspect of the semaphorins is that they can also act as receptors in various processes such as photoreceptor targeting in *Drosophila*, cardiac development in chicken, and thalamic axon guidance in mammals [16].

### 2.4. Ephrins

Since Sperry’s experiments on frog retinal axon projections to the optic tectum [15], deciphering the underlying mechanisms has been the focus of many researchers. Axon from the temporal and nasal part of the retina shows regional preference, respectively, for anterior and posterior parts of the tectum in lower vertebrates and for the lateral geniculate nucleus (LGN) of the thalamus in higher vertebrates. Coculture experiments demonstrated that incubation with membranes from inappropriate region of the tectum causes growth cone collapse in retinal ganglion cells (RGCs) [32]. In addition, incubating the membranes with phosphatidylinositol-specific phospholipase C (PI-PLC) eliminated this effect, which suggested that the causative molecule is GPI-linked [33]. Purification of this molecule led to the discovery of EphrinA5, which is now known to be expressed in an increasing anteroposterior gradient across the tectum [5,16,17].

Since then, two subfamilies of ephrin molecules have been discovered: GPI-anchored EphrinA, represented by two classes and transmembrane EphrinB, represented by three classes. Both subfamilies are unable to elicit a response when they are released from the membrane, suggesting that ephrins are short range repulsive molecules. Class A ephrins signal through class-A Eph receptors, represented by eight members, while class B ephrins signal through class-B Eph receptors, represented by six members, with varying degree of selectivity. As opposed to the EphrinA-EphA pathway, which controls the topographic mapping through the anterior-posterior axis of the tectum (or LGN), EphrinB-EphB pathway is involved in the mapping of the dorsal-ventral axis [5,16,17].

EphrinB can also be involved in reverse signaling upon interacting with EphB receptors [5,16,17], which leads to phosphorylation of a tyrosine residue in the intracellular domain of the Eph tyrosine kinase receptor. Eventually, the same idea was also proven for the class A ephrins, despite the fact that they lack an intracellular domain. It is believed that they act in *cis* manner through coreceptors [17]. Apart from their roles in the topographic mapping, ephrins also function as short-range attractants and repellants in the guidance of many central and peripheral axons, as well as in the pruning of axonal trajectories. Regulation of dendritic morphology and synaptogenesis are also among the functions of ephrins [16].

## 3. Molecular Mechanisms of Axon Guidance

### 3.1. Regulation of Receptor Complexes

As a result of the research addressing the molecular functions of guidance cues and their receptors, a common theme has emerged, which proposes that the receptor complex rather than the guidance cue itself determines the reaction of the growth cone to a given cue [18,34]. Indeed, a wide range of intracellular mechanisms ensures that, in a given moment, the growth cone is well furnished with the correct combination of receptor complexes and is ready to respond to the guidance cues. However, the current knowledge we have about the regulatory mechanisms of the guidance cue/receptor complexes is incomplete as there are more mechanisms to be revealed. Nonetheless, there have been many advances in the elucidation of some of these mechanisms ranging from transcriptional regulation to proteolytic cleavage of the receptor complexes [34].

### 3.2. Trafficking and Surface Enrichment of Guidance Receptors

In theory, regulating the surface expression of a receptor should affect the strength of the signaling through its ligand. Indeed, several in vivo and in vitro studies have hinted at the importance of regulated receptor trafficking in axon guidance. Examples of this level of regulation would be the trafficking of Robo by Comm in flies [34]; trafficking of DCC by protein kinase A (PKA) and its surface enrichment through Hsc70-TrioGEF in rat [34,35]; and trafficking of UNC-40 (DCC), UNC-5, and SAX-3 (Robo) in *C. elegans* [34].

### 3.3. Regulated Endocytosis of Guidance Receptors

Another way of controlling the surface expression of a receptor, in parallel to its trafficking to the cell membrane, is the regulated internalization through endocytosis. Regulated endocytosis of receptor complexes is an integral part of axon guidance. For example, endocytosis of ephrin-Eph ligand/receptor complex has been shown to be important for repulsive responses in mice. In addition, interaction and coendocytosis of receptor neuropilin-1 (Npn-1) with cell adhesion molecules L1 and transient axonal glycoprotein-1 (TAG-1) has been shown to be crucial for Sema3a signaling in mice. Finally, selective internalization of UNC5A, but not DCC, by active protein kinase Cα (PKCα) switches from repulsive netrin-1 signaling to attractive one [34,36].

### 3.4. Proteolytic Processing of Guidance Receptors

Several studies have documented that proteolytic cleavage of guidance cues and their receptors by ADAM metalloproteases and matrix metalloproteases is important for axon guidance in vivo, in both invertebrates and vertebrates. For instance, impairing metalloprotease-dependent ectodomain shedding of DCC results in potentiated netrin-1 signaling. Among these metalloproteases, the Kuzbanian/ADAM10 (kuz/ADAM10) family is of particular interest as they have been linked to the signaling pathways of more than one guidance cues. Studies in *Drosophila* have reported ADAM10 to be a positive regulator of Slit-Robo signaling. Furthermore, cleavage of the ligand ephrin-A2 and the receptor EphB2 by ADAM10 suggests that, in the context of ephrin-Eph signaling, ADAM10 targets both the ligand and the receptor [34,36]. In addition, more evidences are emerging to support a role for ADAM10/gamma-secretase-mediated sequential cleavage of receptors in axon guidance. DCC and a number of ephrins ligands appear to be the targets of a similar mechanism [34,36].

### 3.5. Downstream Signaling from Axon Guidance Receptor Complexes

Upon assembly of the axon guidance cues with their appropriate receptor complexes, a myriad of signaling pathways are exploited to steer the growth cone. Although complete understanding of guidance receptor signaling is currently lacking, several components of downstream signaling pathways have been identified in recent years [34]. Examples of such intracellular components would be calcium, cyclic nucleotides, and Rho GTPases [18,34,36]. Here, we will briefly talk about the involvement of calcium and cyclic nucleotides signaling in axon guidance. However, our main focus will be on the role Rho GTPases and their upstream regulators—GAPs and GEFs—in axon guidance.

#### 3.5.1. Calcium and Cyclic Nucleotides

Based on in vitro studies, calcium and cyclic nucleotides (cAMP and cGMP) can directly mediate guidance receptor signaling and can modulate the response strength, as well. There is an intimate interplay between calcium and cyclic nucleotide signaling in modulation of axon guidance through the mechanisms that involve soluble adenylyl cyclases, nitric oxide synthase (NOS), plasma membrane Ca^2+^ channels, and calcium-induced calcium release (CICR) [37,38].

Overall, chemoattractants such as netrin-1 lead to membrane depolarization, while repellents such as Slit and Sema lead to membrane hyperpolarization by modulating Ca^2+^ influx [39,40,41,42]. As to cyclic nucleotides, their levels or rather ratios—particularly the cAMP to cGMP ratio—determine the growth cone response to guidance cues: high cAMP/cGMP ratio favors attraction and low cAMP/cGMP favors repulsion [43,44,45]. As well, many evidences have indicated that a correct balance of endocytosis and exocytosis can control growth cone turning in response to second messenger signaling [46].

Even though the main bulk of data related to the role of calcium and cyclic nucleotides in axon guidance is based on in vitro studies, more and more data is emerging that supports their importance in vivo, as well [36].

#### 3.5.2. Linking Axon Guidance Receptor Signaling to the Actin Cytoskeleton: Rho GTPases and Their Regulators—GAPs and GEFs

##### The Rho Family of Small GTPases in Guidance Receptor Signaling

In order to mediate the cellular response of the growth cone to its extracellular environment, guidance cue/receptor complexes must alter the growth cone morphology by modulating the local cytoskeleton [34]. The growth cone has three zones based on the type of prevailing cytoskeletal elements: the central zone is occupied mainly by microtubules (MTs), the peripheral zone is occupied mainly by actin or microfilaments (MFs), and finally, the transition zone represents the part of the growth cone, where microtubule and actin filaments overlap [16,47,48].

To respond to the guidance cues, the growth cone has to maintain proper dynamics of both actin and microtubule cytoskeletons. In support of this, drugs such as nocodazole and latrunculin, which inhibit microtubule and actin polymerization, respectively, cause unidirectional axon outgrowth. This data suggests that the dynamic nature of both cytoskeletal elements is crucial for changing the growth cone direction [47]. Despite the equal importance of both cytoskeletal elements, actin is the primary target of guidance cue/receptor signaling as it predominates at the leading edge—the most motile part of the growth cone. At the leading edge, actin can form distinct type of superstructures: finger-like structures such as filopodia, and web-like structures as lamellipodia [2,16,34,48,49,50]. During growth cone turning, actin dynamics at the leading edge are exploited to direct the advance of microtubule [48]. The attractive cues promote actin polymerization at the tip of the growth cone, while the repulsive cues decrease it to produce turning effect towards or away from the source of a given cue, respectively [51]. In this regard, filopodia is particularly important as asymmetric filopodial growth is the starting point for the turning of the whole growth cone.

Although it is not fully understood how guidance cue/receptors signaling controls actin dynamics, it has been widely documented that they regulate, directly or indirectly, the activity of Rho family of small GTPases [34,36,48,49,51]. The Rho GTPases, in particular the well-studied members RhoA, Rac1, and Cdc42, are known for their roles in cell motility and regulation of cytoskeletal structures [52]. The family contains well-studied members RhoA, Rac1, and Cdc42 along with some other relatively less-studied members. Pioneering work in fibroblasts have demonstrated that activation of RhoA, Rac1, and Cdc42 leads to formation of distinct actin-based structures—stress fibers, lamellipodia, and filopodia, respectively [52,53,54].

**Figure 2:**
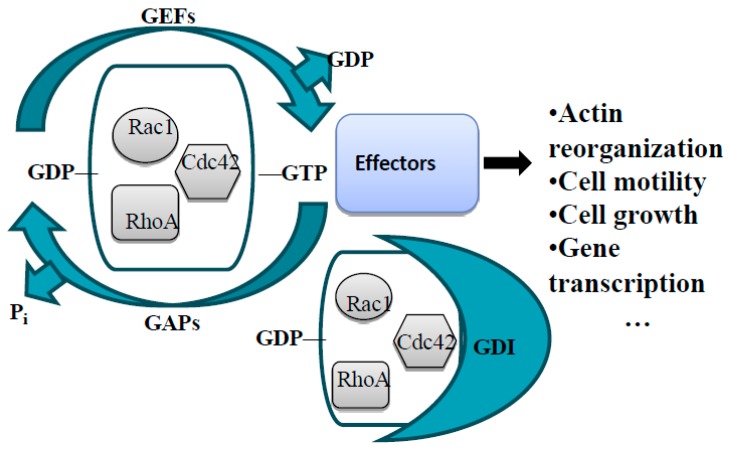
The mechanism of regulation of Rho GTPases. The Rho family of small GTPases shuttle between active, GTP-bound, and inactive, GDP-bound, states. This cycle is coordinated by three classes of regulatory proteins: (1) GEFs (Guanine nucleotide-exchange factors), which activate the small GTPases by performing the GDP to GTP exchange; (2) GAPs (GTPase-activating proteins), which inactivate the small GTPases by enhancing their intrinsic enzymatic activity to hydrolyze the GTP into GDP; and (3) GDIs (Guanine nucleotide-dissociation inhibitors), which sequester the small GTPases in their GDP-bound, inactive state in the cytosol.

Even though it was originally thought that the attractive cues induce leading edge actin polymerization through Rac1 and Cdc42 activation and the repulsive cues induce retraction through RhoA activation, further research in the field revealed that the real case is far more complex than this [36]. Work in fibroblasts, rat commissural neurons, and N1E-115 neuroblastoma cells showed that netrin-1/DCC signaling increases Rac1 and Cdc42 activities, while decreasing RhoA activity [55,56,57] (Figure 1). Surprisingly, as opposed to its well reported repulsive role [30,58], overexpression of Unc5a in N1E-115 neuroblastoma cells has been shown to induce neurite outgrowth by increasing Rac1 and Cdc42 activity [59]. Moreover, it has also been reported that Unc5a-overexpressing N1E-115 neuroblastoma cells lead to transient Rac1 activation in early stages and RhoA activation in the later stages of neurite outgrowth [59]. Upon Sema stimulation, plexin-B1 directly interacts and sequesters active Rac1, along with activation of RhoA [60,61]. However, Sema3A-plexin-A signaling activates Rac1 [62] (Figure 1). Slit-Robo signaling causes decreased Cdc42 activity and increased RhoA and Rac1 activity [63,64]. Finally, ephrin-Eph forward signaling causes increased RhoA activity, but it also leads to transient decrease of Rac1 activity in retinal ganglion cells (RGCs) [65,66], while Eph-ephrin reverse signaling activates Rac1 and Cdc42 [67]. As shown in these examples, there is no solid pattern of Rho GTPase signaling downstream of the guidance receptors (Figure 1) and further research is required to fully comprehend their role in axon guidance [34,36,49]. The first step toward this end can be to understand how the guidance receptor complexes lead to activation or inhibition of the Rho GTPases. Except for the case of Plexin-B1, guidance receptors do not interact with Rho GTPases directly, but rather deploy signaling adaptors and kinases to modulate the activity of their upstream regulators [34]. Rho GTPases exist in two states: active, GTP-bound, and inactive, GDP-bound. In their active state, Rho GTPases interact with their downstream effectors and modulate their cellular functions. There are three groups of upstream regulators that control the activity of Rho GTPases: GTPase-activating proteins (GAPs) stimulate the intrinsic low GTPase activity of Rho GTPases and act as negative regulators by switching the small GTPases from GTP-bound to GDP-bound state; guanine nucleotide exchange factors (GEFs) act as positive regulators by replacing the GDP with GTP, therefore activating the small GTPases; finally, guanine nucleotide dissociation inhibitors (GDIs) bind GDP-bound form of the small GTPases and sequest them in the cytoplasm, which inhibits the dissociation of GDP and keeps the small GTPases in the inactive state [49,68,69] (Figure 2).

##### General Information about GEFs and GAPs

The first GAP protein—p50RhoGAP (ARHGAP1)—was identified almost thirty years ago, in 1989, from human spleen extract [70]. The first GEF protein—MCF-2/DBL (ARHGEF21)—, on the other hand, was identified four years earlier than that, in 1984, as an oncogene, using the NIH3T3 mouse fibroblast focus formation assay [71]. Since then, about 80 RhoGAPs and 82 RhoGEFs have been identified for 20 Rho GTPases in eukaryotes [51,72,73]. In all species, GAPs and GEFs outnumber Rho GTPases, which can be rationalized with four possible explanations: (1) despite the ubiquitous expression pattern of most GAPs/GEFs, some of them show tissue specificity; (2) some GAPs/GEFs are specific only for one member of the Rho GTPases, while the others are active towards multiple members, at least in vitro; (3) each GAP/GEF might be involved in specific RhoGTP-driven signaling pathways; (4) in addition to their conventional roles, GAPs/GEFs might act as scaffold proteins to mediate cross-talk between different GTPase pathways or the formation of protein complexes. Most likely, a combination of more than one of these possibilities is underlying the actual explanation [74].

There are two classes of RhoGEFs: diffuse B-cell lymphoma (DBL) and dedicator of cytokinesis (DOCK) families of proteins. There are 71 identified DBL GEFs, the classical GEFs, with characteristic DBL homology (DH) and pleckstrin homology (PH) domains. The DH domain catalyzes the GDP-GTP exchange, while the PH domain serves different functions such as localization to the plasma membrane, interaction with cytoskeletal proteins, and regulation of the DH catalytic activity. The DOCK family, however, is represented by 11 members, which contain DOCK-homology regions (DHR) 1 and 2. The DHR1 domain is involved in membrane localization and the DHR2 domain catalyzes the GDP-GTP exchange [51,71,72,73,75,76,77,78,79]. Another feature that distinguishes DOCK GEFs from DBL GEFs is that unlike DBL GEFs, DOCK GEFs activates only Rac1 and/or Cdc42, but not RhoA or the other members of Rho GTPases [76,79]. RhoGAPs, on the other hand, are characterized by the presence of a conserved RhoGAP domain [74,75]. In the current terminology RhoGAPs and DBL family of RhoGEFs are assigned a new, standardized name: Rho GTPase activating proteins (ARHGAPs) and Rho guanine nucleotide exchange factors (ARHGEFs), respectively. Both RhoGAPs and RhoGEFs are multidomain proteins with several lipid and/or protein interacting domains, which mediate their subcellular localization or formation of protein complexes. In addition, combination of different GAP and/or GEF domains can be found in a single GAP or GEF protein. This probably serves the purpose of linking different GTPase signaling pathways [74,75]. For more in-depth information about RhoGEFs and GAPs, their evolution, working mechanisms, and involvement in diseases we refer the readers to other review articles [51,71,72,73,74,75,76,77,78,79,80].

##### RhoGAPs and GEFs in Axon Guidance

As the direct regulators of Rho GTPases, RhoGAPs and GEFs have been demonstrated to be implicated in axon guidance and pertaining signaling mechanisms [34,35,36,48,49,51,81,82]. It is very cumbersome to identify the specific GAPs and GEFs that are involved in guidance receptor signaling pathways because of the following reasons: (1) GAPs/GEFs often show redundancy in their roles, (2) Individual GAPs/GEFs might be linked to several signaling pathways, (3) GAPs/GEFs generally participate only in part of a given pathway [34]. However, work done using cultured cells and in vivo animal models in past years have revealed several GAPs and GEFs as integral part of guidance receptor signaling [34,35,36,48,49,51,81,82]. For example, mice lacking a Rho-specific GAP protein with high expression in the nervous system, p190RhoGAP (ARHGAP35), show clear guidance defects in the axonal projections of the posterior limb of the anterior commissure [83]. Here, we will briefly overview the well-established roles of RhoGAPs and GEFs in axon guidance, focusing particularly downstream of the four classical guidance cues and their receptor complexes (Figure 1 and Table 1).

### 3.6. Netrin-1/DCC

Recent studies have delineated two GEFs as Rac1 activators downstream of the netrin-1/DCC signaling pathways in axon outgrowth and guidance: Trio (ARHGEF23) and DOCK180 (DOCK1) GEFs [35,81,84,85,86]

Trio harbors two GEF domains: one is active toward Rac1 and RhoG and the second one is active for RhoA [100]. Trio is a positive contributor of the embryonic *Drosophila* CNS and it can physically interact with both Frazzled and DCC [34,36,49,84]. The work by DeGeer et al. has reported that Trio, together with the Hsc70 chaperone, mediates both Netrin-1/DCC-driven axon outgrowth and DCC surface expression in the growth cone of embryonic rat cortical neurons [35]. Furthermore, Trio^−/−^ embryonic brains do not show netrin-1-induced Rac1 activation and Trio-deficient mice display guidance defects similar to those of the DCC^−/−^ mice [84]. However, Trio cannot be the only GEF that is responsible for the netrin-1/DCC signaling as Trio^−/−^ mice show milder commissural axon guidance defects than DCC^−/−^ mice [36,84].

DOCK180 is another GEF to be involved in netrin-1/DCC signaling. Like Trio, it interacts with DCC and activates Rac1. DOCK180 has been reported to participate in vitro in netrin-1/DCC-induced Rac1 mediated attraction, outgrowth, and turning of mouse cortical and commissural neurons. Its knockdown in the chick spinal cord leads to reduced midline crossing of commissural neurons [86]. However, commissural or cortical axon guidance defects in DOCK180 null mice have not been reported yet. Whether these two GEFs act in the same or parallel pathways is still not clear [34,36,49,51].

### 3.7. Slit-Robo

The Slit/Robo-induced Rac1 activation provided an evidence for the fact that Rac activation can also contribute to the repulsive responses [63,64,96]. In *Drosophila*, Slit/Robo signaling and Rac activity is linked by specific RacGAP and GEF proteins. The evolutionary conserved RacGAP, vilse/CrGAP (ARHGAP39) is involved in slit-mediated midline repulsion of CNS axons. Interestingly, both increased and reduced levels of vilse/CrGAP causes dosage-dependent defects in slit/robo-induced repulsion, which indicates the importance of precise modulation of vilse/CrGAP activity [96,101]. However, there should be other Rac regulators involved in slit/robo pathway as vilse mutants display only minor midline crossing defects [36]. Sos—a dual GEF with activity towards both Ras and Rho GTPases—is a promising candidate in that sense based on its expression in the *Drosophila* CNS, its genetic interaction with *slit* and *robo* mutants [97], and its interaction with the *Drosophila* ortholog of Nck adaptor protein—Dock [63]. Work using cultured human 293T cells has reported that upon Slit stimulation, Sos translocates to the plasma membrane, where it interacts with Robo and induces lamellipodia formation [98].

In line with the finding that slit/robo signaling leads to decreased Cdc42 activity [63,64], Wu et al. [99] have reported that, upon its interaction with slit, robo interacts with and activates Slit-Robo Rho GTPase activating protein 1 (SrGAP1/ARHGAP13), which in turn inactivates Cdc42. The authors have reported that robo1 show tissue specific colocalization with SrGAP1 and 2 in the anterior subventricular zone (SVZa) of neonatal mice. Moreover, they have shown that SrGAP1 coimmunoprecipitates with robo1 from rat neocortical extracts. Further experiments using truncated SrGAP1 proteins have shown that the SH3 domain of SrGAP1 is sufficient to interact with the proline rich CC3 motif of robo1. In support of these data, using yeast two-hybrid system, Wong et al. [64] have demonstrated that the intracellular domain of the rat robo1 interacts strongly with SrGAP1, 2 and 3.

### 3.8. Sema-Plexin

In *Drosophila*, upon contact with Semaphorins, Plexin-Bs prevent the interaction of Rac with its effector by binding and sequestering active Rac [60]. Also, work from cultured cells suggests that active Rac increases both Plexin-B1 affinity towards Sema4D and its localization to the cell surface [102]. Meanwhile, Sema4D/Plexin-B1 signaling induces RhoA activity through two RhoGEFs—PDZ-RhoGEF (ARHGEF11) and Leukemia-associated RhoGEF (LARG/ARHGEF12) [60,61]. Both of these GEFs interact directly with the PDZ-binding domain of Plexin-B1. Dominant-negative (DN) versions of PDZ-RhoGEF and LARG abolish Sema4D-induced growth cone collapse in hippocampal neurons [61].

Unlike Plexin-Bs, Plexin-A induces Rac1 activation, to mediate growth cone collapse [103]. Work in cultured chick dorsal root ganglion (DRG) neurons has reported that Sema3A signaling through Plexin-A/Npn-1 leads to suppression of neurite outgrowth [94]. Further examination of the downstream pathway has shown that the processes requires Rac1 activation through a FERM domain-containing GEF—FERM, RhoGEF, and pleckstrin domain-containing protein 2 (FARP2). FARP2 is normally associated with PlexinA1/Npn-1 and it is released upon Sema3A stimulation. This release activates the GEF activity of FARP2 and leads to elevated Rac1 activity.

The first GAP that has been implicated in the sema/plexin signaling pathway is p190RhoGAP. Barberis and colleagues [95] have reported that p190RhoGAP mediates Sema4D/PlexinB1-induced neurite outgrowth in PC12 neuroblasts. They also report that p190RhoGAP plays role downstream of PlexinB1 signaling in various cell types such as fibroblasts, tumor epithelial, and primary endothelial cells. Although this finding contradicts the mainly accepted idea that Sema4D/PlexinB1 signaling leads to RhoGEF-mediated, RhoA activation, the authors claim that the two processes occur in temporally different manners [95]. Although they contribute to the formation of a more complete picture, the importance of these findings in vivo has yet to be validated [36].

### 3.9. Ephrin-Eph

Mutations in either of the genes encoding ephrinB3 or the receptor EphA4 lead to misrouting of interneuron axons of the mouse locomotor central pattern generator (CPG), the circuit necessary for coordinating alternating limb movement [87,88,89,90,91,104]. This subsequently leads to a particular phenotype called hopping gait [104]. The phenotype originates from defective ephrin-Eph forward signaling [89]. Interestingly, mutations in a gene encoding a Rac-specific GAP, α2-chimaerin result in an almost identical phenotype, hinting at the importance of α2-chimaerin-regulated Rac1 activity in ephrin-Eph forward signaling [87,90,91]. α2-chimaerin contains two domains that interact with EphA4: a N-terminal SH2 domain that binds to a phosphorylated juxtamembrane tyrosine residue of EphA4 and a C-terminal region that binds EphA4 constitutively. EphA4 mediates ephrinB3-induced tyrosine phosphorylation of α2-chimaerin, which increases its GAP activity towards Rac1 [90].

Surprisingly, the restoration of Rac1 activity also seems to be crucial for ephrin/Eph-driven growth cone collapse, particularly for class-B ephrin/Eph signaling [65,105,106]. It seems that Rac1 activity-dependent endocytosis of ephrin/Eph complexes in *trans* to neighboring cells is an integral part of their repulsive mechanisms [107,108]. Members of the Vav family of DBL GEFs—Vav2 and Vav3—have been reported to mediate Rac1-dependent endocytosis of ephrin/Eph ligand–receptor complexes [92].

Ephrin/Eph signaling also leads to RhoA activation through DBL family RhoGEFs—ephexins. Two members of ephexin, ephexin1/NGEF (ARHGEF27) and ephexin5/VSM-RhoGEF (ARHGEF15), are expressed in the mouse brain [93]. Depletion of mouse ephexin1 and chick ortholog c-ephexin leads to defects in ephrinA-induced growth cone collapse and axon repulsion, as well as defects in axon outgrowth [93].

In parallel to the forward signaling, ephrinB can behave as a receptor and induce reverse signaling upon contacting cognate Ephs [109]. DOCK180 has also been implicated in ephrinB3-driven reverse signaling in stereotyped pruning of exuberant mossy fiber axons in the hippocampus. Nck2 adapter protein appears to link ephrinB3 to DOCK180-mediated activation of Rac1 and Cdc42, which subsequently leads to axon retraction [67].

## 4. Regulators of Rho GTPases in Neurological Disorders

In this section, we will overview the GAPs and GEFs that are involved or genetically associated to neurological diseases and disorders. We will focus mainly on the work published from mammalian research, in particular from human cases (Table 2). We will only review the DBL family of GEFs as the implication of the DOCK family of GEFs in neurological diseases has recently been reviewed elsewhere [79].

### 4.1. RhoGAPs

#### 4.1.1. ARHGAP2 (α-Chimaerin/CHN1)

The chimaerin subfamily of RhoGAPs has Rac1-specific GAP activity. The subfamily contains five members (α1-, α2-, β1-, β2-, and β3-chimaerins), all of which are the results of alternative splicing of two genes, *ARHGAP2 (CHN1)* and *ARHGAP3 (CHN2)*. By controlling Rac1 activity, α1-chimaerin plays a crucial role in the regulation of dendritic growth during neuronal development [111,161,179]. By comparing the expression levels of α1- and α2-chimaerins in postmortem brains of individuals with Alzheimer’s disease (AD) or unaffected individuals, Kato et al. [161] have demonstrated that α1-chimaerin mRNA levels are significantly reduced in the temporal lobe of the AD patients in comparison to brains of unaffected individuals. However, no significant difference has been observed in the levels of α2-chimaerin expression between the two groups.

α2-chimaerin has been implicated in axon guidance and is highly expressed in developing ocular motor neurons in rats and mice [87,88,90,168]. Two related studies [168,169], have demonstrated that several missense mutations leading to enhanced dimerization and Rac1-GAP activity of α2-chimaerin causes Duane’s retraction syndrome (DRS) with strabismus phenotype. DRS is a congenital eye movement disorder caused by defects in the innervation of extraocular muscles by the axons of brainstem motor neurons. In support of this, a study by Ferrario and colleagues [170] has shown that Semaphorin 3A and 3C (Sema3A/C)/PlexinA signaling acts upstream of α2-chimaerin to modulate the axon guidance of ocular motor neurons. Importantly, implications of α2-chimaerin in CNS is not limited to DRS. Using global and conditional knock-out mouse models, Iwata et al. [111] have demonstrated that deletion of α2-chimaerin in early development leads to defects in contextual fear learning. The authors have also shown that this effect is not observed if α2-chimaerin is deleted during adulthood, which suggests that α2-chimaerin acts during development to establish adulthood cognitive abilities.

#### 4.1.2. ARHGAP15

ARHGAP15 is a Rac-specific GAP protein. ARHGAP15 is expressed in both excitatory and inhibitory neurons of the adult hippocampus [112]. In a recent study by Zamboni and colleagues [112], ARHGAP15 has been associated with cognitive defects in mice. The authors have shown that loss of ARHGAP15 causes increased Rac1/3 activity, which leads to defects in the directionality and efficiency of migration of inhibitory neurons. These defects caused a reduction in numbers of inhibitory neurons and, subsequently, an alteration of the balance between inhibitory and excitatory synapses in favor of the latter, in hippocampus. As a result of the aforementioned changes, defects in hippocampus-dependent functions, such as working and associative memories, were observed in adult ARHGAP15^−/−^ mice. The study stresses the importance of Rac activity and its precise regulation in developing hippocampal neurons.

#### 4.1.3. ARHGAP18 (MacGAP, SENEX)

ARHGAP18 is a RhoA and RhoC-specific GAP protein [180]. In two related publications [118,119], authors have used functional Magnetic Resonance Imaging (fMRI) of the dorsolateral prefrontal cortex (DLPFC) and genome-wide association study (GWAS) to identify novel schizophrenia-associated genes. As a result, they have identified *ARHGAP18* as a schizophrenia associated gene. Similar results have recently been published by Guo et al., showing an association between *ARHGAP18* polymorphisms and schizophrenia in the Chinese-Han population [120].

#### 4.1.4. ARHGAP28

ARHGAP28 is a RhoA-specific GAP protein [181]. In a recent study by Jiang et al. [127], a point mutation in *ARHGAP28* gene has been associated to the most common type of migraines—migraine without aura (MWO). Migraine is a group of recurrent headache disorder that is clinically characterized with neuropsychiatric aspects. Authors have targeted a cohort of 8 individuals—4 with MWO and 4 without MWO. In 4 individuals with MWO, authors have detected mutations in six genes, one of which is *ARHGAP28.* A missense mutation in the *ARHGAP28* gene, which converts the threonine 31 residue to serine (T31S), reduced the ARHGAP28 expression, elevated RhoA and ROCK activities, resulting in cerebral vasoconstriction, spasm, and migraine, as well as an increase in the intensity of inflammatory reaction in the brain [127].

#### 4.1.5. ARHGAP32 (p250GAP)

ARHGAP32 is a GAP protein with GAP activity towards Cdc42, Rac1, and RhoA [182,183]. It is highly enriched in the CNS, where it is the primary target of the neuronal-specific microRNA—miR132. miR132 promotes neurite outgrowth by inhibiting ARHGAP32 expression [184]. A recent study [128] exploiting the mouse model of the Huntington’s disease (HD) has shown that miR132 levels in this model is severely reduced, leading to increased expression of its primary target—ARHGAP32. HD is a dominant-inherited disease caused by the expansion of a CAG repeat in the *huntingtin (htt)* gene. The disease is currently incurable and eventually fatal. This study suggests that the cellular level of ARHGAP32 is important for proper brain development [128].

#### 4.1.6. ARHGAP14 (srGAP3) and ARHGAP34 (srGAP2)

ARHGAP34 and ARHGAP14, better known as srGAP2 and srGAP3, respectively, are members of the Slit-Robo GTPase-activating protein subfamily (SrGAP) of RhoGAP with Rac1-specific GAP activity [138,167,185]. SrGAP2 is expressed in the entire developing cortex [186]. It prevents neuronal migration and promotes neurite outgrowth and neurite branching. SrGAP has also been shown to interact with robo1 and to regulate Cdc42 activity in a slit-robo-dependent manner [64,99]. A study by Saitsu and colleagues [167] has shown that a balanced translocation mutation that disrupts the *srGAP2* gene is associated with early infantile epileptic encephalopathy. SrGAP3 is highly expressed in the cortex and hippocampus, structures with crucial importance in the higher cognitive functions. It has been reported that loss of one of the *srGAP3* alleles as a result of a translocation mutation is associated with mental retardation (MR) [139]. In a more recent study [138], SrGAP3 has been demonstrated to be involved in spine development and its loss has been implicated in disruption of long-term memory.

#### 4.1.7. ARHGAP33 (TCGAP)

ARHGAP33 is a Cdc42-specific GAP protein that is prominently expressed in developing and mature brain. It is involved in neurite outgrowth and branching as well as dendrite arborization and morphology [187,188]. In two independent studies, ARHGAP33 has been implicated in neuropsychiatric developmental disorders, such as autism spectrum disorders (ASDs) and schizophrenia. Schuster et al. [121] have demonstrated that ARHGAP33 regulates dendritic spine maturation, synaptic transmission, and social behavior in mice, in a gender-specific manner. The study shows that loss of ARHGAP33 causes autism-like alteration in social behavior. In a more recent study [122], ARHGAP33-deficient mice show impaired dendritic spine morphology in the hippocampus, which leads to several behavioral defects such as working memory, learning, habituation, and anxiety, similar to the ones that are observed in neuropsychiatric developmental disorders [189,190]. Using immortalized human lymphocytes, the authors showed that the expression level of ARHGAP33 was lower in 45 schizophrenia patients in comparison to 45 sex/age-matched controls. They also showed that the genetic variation in the *ARHGAP33* gene is associated with Schizophrenia. In line with these data, the authors have demonstrated that polymorphisms in *ARHGAP33* gene may be linked to several schizophrenia related vulnerabilities in the brain morphology [122].

#### 4.1.8. ARHGAP41 (Oligophrenin-1/OPHN1)

ARHGAP41, better known as oligophrenin-1 (OPHN1), is a RhoGAP highly expressed in the brain. It was first identified as a gene mutated in patients with X-linked mental retardation (XLMR) [140,141,142]. Mental retardation (MR), recently renamed as intellectual disability (ID), is an early onset condition with subaverage intellectual functioning (IQ < 70). It is defined as a nonprogressive reduction in cognitive abilities [140,142,191]. Oligophrenin-1 was shown to have GAP activity towards RhoA, Rac1, and Cdc42 [141]. Studies on more XLMR cases by different research groups have proven that different deletion and duplications in the *OPHN1* gene are causative of X-linked mental retardation with a myriad of clinical and morphological phenotypes such as epilepsy, rostral ventricular enlargement, cerebellar hypoplasia, neonatal hypotonia, early onset seizures, marked strabismus, and language impairments [140,141,142,143,144,145].

#### 4.1.9. ARHGAP43 (SH3BP1/3BP-1)

RHGAP43, better known as SH3 domain binding protein 1 (SH3BP1), is a Rac1, Cdc42, and TC10-specific GAP protein [162,192]. *SH3BP1* gene resides upstream of the gene encoding haloacid dehydrogenase family phosphatase chronophin (CIN/PDXP) [162]. Recently, an mRNA transcript that is a partial fusion of SH3BP1 and CIN gene products has been linked to Alzheimer’s disease (AD) [162]. The authors have named this mRNA and the protein it encodes BARGIN or BGIN. The study demonstrates that the C-terminal end of BGIN noncovalently binds to poly-ubiquitin, which promotes its membrane localization leading to the inactivation of Rac1. In addition, the authors have observed BGIN-Ub interaction in tangled aggregates in AD brain. AD is caused by the accumulation of ß-amyloid (Aß) plaques and is the leading cause of dementia. Finally, using an amyloid precursor protein (APP) model, they have documented that BGIN mediates, at least partially, Rac1 inhibition and reactive oxygen species (ROS) generation.

#### 4.1.10. ARHGAP44 (RICH2/Nadrin1)

ARHGAP44 is a Rac1 and Cdc42-specific GAP protein. It has important role in the CNS during dendritic spine morphogenesis. It was first identified as an interacting partner of SHANK3—a SHANK family member that coordinates structural and functional changes in postsynaptic compartments [130,193]. Since SHANK family has been closely associated with ASD and schizophrenia, its interaction with SHANK3 suggests an involvement of ARHGAP44 in neuropsychiatric diseases as well. To address this, Sarowar et al. have created an ARHGAP44-depleted mouse line [130]. They have shown that depletion of ARHGAP44 leads to increase in the spine volume and the number of spines with multiple heads. At the behavioral level, the authors have observed ASD-like traits such as stereotypic behavior, specific phobia, and abnormal motor behavior [130].

#### 4.1.11. ARAP1, ARAP3, ARHGAP12, ARHGAP29, ARHGAP40, and ARHGAP45 (HMHA1)

By analyzing the gene expression profile of skin fibroblasts obtained from either healthy individuals or individuals with bipolar disorder (BD), Logotheti et al. [110] identified that genes involved in small GTPases-mediated signal transduction are downregulated. Among these genes, they mention ARAP1 and 3, ARHGAP12, 29, 40, and Minor Histocompatibility Antigen HA-1 (HMHA1/ARHGAP45), all of which are GAPs for the Rho GTPases. ARAP (ArfGAP and RhoGAP with ankyrin repeat and PH domains) subfamily contains both ArfGAP and RhoGAP domains, which allow them to regulate both families of small GTPases [194,195]. HMHA1 is a cancer therapeutic target with a RhoGAP activity [196,197]. ARHGAP12 and 29 are ubiquitously expressed and they carry GAP activities towards Rac1 and RhoA, respectively [198,199,200].

#### 4.1.12. ARHGAP46 (GMIP)

ARHGAP46, better known as GEM-interacting protein (GMIP), is a ubiquitously expressed protein with RhoA-specific GAP activity. It is crucial for neurite outgrowth, axon guidance, neuronal migration, and synaptic functions [137,201]. Tadokoro et al., [137] have shown that ARHGAP46-related SNPs are associated with major depressive disorder (MDD) in Japanese population, particularly in male subjects.

#### 4.1.13. Myosin IXb (MYO9B)

MYO9B is a RhoA-specific GAP protein that has been associated with autoimmune disease—celiac disease [123,202]. In their search for a candidate gene that could fit the long-thought association between autoimmune diseases and schizophrenia, Jungerius and colleagues [123] have tested the hypothesis that MYO9B is the candidate gene. Interestingly, they show strong correlation between MYO9B associated SNPs and schizophrenia.

#### 4.1.14. Ral Binding Protein 1 (RalBP1/RLIP76)

RalBP1 is a Rac1 and Cdc42-specific GAP protein. It also has GAP activity towards the Ras-like GTPase (Ral), which renders RalBP1 crucial for crosstalk of Ras-Rho signaling [159,203]. Interestingly, it can also activate Rac1 in a GTP-R-Ras-dependent and Ral-independent manner, through Arf6 GTPase activity [204]. In forebrain, hippocampal CA1 neurons show the highest expression level of RalBP1. Using mouse RalBP1 hypomorphs—RalBP1 expression is only 18% of the WT levels—, Bae et al. showed that, although it does not cause seizures per se, reduction in the RalBP1 levels renders mice more susceptible to seizures by reducing the seizure threshold [159]. In addition, more data are emerging, which supports a role for RalBP1 as a mediator of pharmacoresistance in the CNS but further research is required in order to substantiate these data [205].

### 4.2. RhoGEFs

#### 4.2.1. ARHGEF2 (GEF-H1)

ARHGEF2, better known as GEF-H1, is a microtubule-associated RhoA-specific GEF protein. It links actin and microtubule dynamics. ARHGEF2 is involved in mitotic spindle formation and orientation [129]. It is expressed by neural precursors and immature neurons in mouse neocortex. ARHGEF2 plays important roles in the CNS in processes ranging from axonal renetworking, dendritic spine retraction, general gene expression, neurogenesis to neural tube closure by mediating RhoA activity [129,146,206]. A case study [146] has demonstrated that a loss-of-function mutation leading to truncated ARHGEF2 protein causes intellectual disability (ID), mild microcephaly, and midbrain-hindbrain malformations. Authors have also demonstrated that mice lacking ARHGEF2 show significant reduction in the volume of the total brain size (microencephaly), the cerebellum, and the brain stem as well as absence of pontine nuclei. These defects recapitulate the phenotypes that have been observed in the human patients and propose a conserved role for ARHGEF2 in humans and mice. In another study, Varma et al. [129] have demonstrated that ARHGEF2-mediated RhoA activation protects rat striatal neuronal cells against MT depolymerizing agents. Interestingly, this rescue phenomenon has been observed only when the neuronal cells express the mutant version of the Htt protein, but not the wild type (WT) version. Considering that the mutation in *Htt* gene leads to Huntington’s disease (HD), this study suggests a possible neuroprotective role for the ARHGEF2-mediated modulation of RhoA activity in HD.

#### 4.2.2. ARHGEF6 (αPIX/Cool-2)

ARHGEF6 is a Rac1/Cdc42-specific GEF protein. It is ubiquitously expressed in different tissues [147,207]. In brain, ARHGEF6 has been shown to be expressed in hippocampal CA1-CA3 cells layer and in cultured neurons it has been shown to colocalize with PSD95 protein at postsynaptic densities of excitatory synapses [208]. In the CNS, ARHGEF6 plays a role in axonal and dendritic branching, regulation of spine morphogenesis, and synapse formation [147]. Loss of ARHGEF6 in mice leads to reduced active Rac1 and Cdc42 levels, altered dendritic morphology, decreased long-term potentiation (LTP), increased long-term depression (LDP), and several behavioral abnormalities, such as navigation errors, disinhibited object exploration, and impairment of complex positional learning [147]. Also, *ARHGEF6* is one of the three RhoGEF encoding genes to be involved in XLMR along with *faciogenital dysplasia protein 1* (*FGD1*) and *ARHGEF9* (*collybistin*) [147,148].

#### 4.2.3. ARHGEF9 (Collybistin)

ARHGEF9, also known as collybistin, is a Cdc42-specific GEF protein with high expression in the developing and adult brain [160]. ARHGEF9 has been shown to mediate the translocation of gephyrin to the postsynaptic membrane microaggregates, which in turn regulates clustering of the receptors for inhibitory neurotransmitters—glycine and γ-Aminobutyric acid (GABA) [209]. Therefore, ARHGEF9 is an important component of the inhibitory synapses [160]. Several case studies have revealed mutations in the *ARHGEF9* gene leading to XLMR, ASD, along with phenotypes such as seizures and epilepsy [131,149,150,151,152,153,160]. A dominant-negative missense mutation, p.G55A, has been linked to severe mental retardation, hyperekplexia, drug-resistant seizures, and premature death in a male patient [160]. A separate study [153] identified a microdeletion and a nonsense mutation in *ARHGEF9* gene causing loss-of-function in two male individuals. The common symptoms for both patients were mental retardation (MR) and epilepsy. Moreover, a missense mutation in *ARHGEF9* gene, p.R338W, has been identified, which causes structural disruption of the ARHGEF9 protein, in several members of a family with XLMR, macrocephaly, and macro-orchidism [151]. Yet another missense mutation was identified by carrying out genetic analysis on an Ethiopian-Jewish family with four affected male individuals, the authors have found a p.G323R mutation. All the affected individuals had intellectual disability, focal epilepsy, and febrile seizures [150]. However, ARHGEF9 mutations are not limited to point mutations: two chromosomal translocation mutations in two different female individuals have been reported. In one of the cases, the mutation led to epilepsy, anxiety, aggression, and MR, while in the other case, the patient had XLMR and sensory hyperarousal [149,152]. Finally, an 82 kb deletion including *ARHGEF9* gene has been detected in an 8-year-old female with ASD, intellectual disability, and speech delay [131]. Altogether, these case studies highlight that the role of ARHGEF9 in gephyrin mediated postsynaptic membrane organization in inhibitory synapses is of critical importance during brain development.

#### 4.2.4. ARHGEF10

ARHGEF10 is a RhoA-specific GEF protein [174]. It is expressed in wide range of tissues with relatively high expression in the spinal cord and dorsal root ganglion. ARHGEF10 has been shown to be involved in neuronal growth, axonal guidance, and to be implicated in human hypomyelination [175]. A missense mutation in the exon 3 of the *ARHGEF10* gene has been shown to cause nonclinical slowed nerve-conduction velocity. The mutation has shown to be inherited in an autosomal dominant fashion and to cause increased GEF activity [176]. In addition, *ARHGEF10* gene has been shown to be associated with Charcot-Marie-Tooth (CMT) disease, a heterogeneous mix of hereditary motor and sensory neuropathies in humans [174,177]. To check the association between CMT genes and chemotherapy-induced peripheral neuropathy (CIPN), Beutler and colleagues have studied 49 CMT genes in a large cohort of cancer patients. During the study, the patients have been administered paclitaxel, a chemotherapy agent. The authors have identified three nonsynonymous SNP in *ARHGEF10* gene to be highly associated with CIPN, one of them showing the strongest association with neuroprotective effect [174]. This study has been successfully replicated in an independent cohort of patients [177]. Another mutation in the *ARHGEF10* gene, a deletion mutation leading to the loss of 50% of its protein product, has been shown to be associated with juvenile-onset inherited polyneuropathy in Leonberger and Saint Bernard dogs. The affected dogs had severe symptoms such as axonal degeneration with progressive clinical signs of weakness and muscle atrophy leading to decreased nerve fiber density and chronic nerve fiber loss [175].

#### 4.2.5. ARHGEF13 (A-Kinase Anchor Protein 13 (AKAP13)/LBC/BRX)

ARHGEF13, better known as AKAP13, is a Rho-specific GEF protein that belongs to A-kinase anchor protein family (AKAP). This family is composed of more than 50 proteins encoded by 10 *AKAP* genes. These proteins share the ability of linking protein kinase A (PKA) to its targets. Recently, using bioinformatics and extensive manual literature mining, Poelmans et al. [132] have demonstrated that AKAPs can be candidate drug targets for the treatment of autism spectrum disorders (ASDs). By analyzing the results of 6 previously published GWASs and emerging signaling networks related to ASDs, they have shown that AKAPs are integrating signaling cascades within and between these networks. AKAP13 positively regulates RhoA, a small GTPase that is involved in neurite outgrowth, regulation of synaptic networks, and modulation of the effect of risperidone treatment for ASDs. In addition, *AKAP13* gene has been found in copy number variations (CNVs) in ASDs patients and its mRNA product is a target of ASDs related microRNAs. AKAP13 is also involved in two ASD-implicated biological processes—innate immunity and melatonin synthesis. Overall, these findings reveal AKAP13 as a promising candidate for ASD-targeted drug development.

#### 4.2.6. ARHGEF23 (Trio)

ARHGEF23, better known as Trio, is a large, multidomain protein with two separate GEF domains—GEF1 and GEF2 [100]. The GEF1 domains is active towards Rac1 and RhoG, while the GEF2 domain is active towards RhoA, which renders Trio a crucial signaling component in crosslinking RhoA-Rac-mediated pathways [154,155,157]. Trio is highly expressed in the developing brain of rodents and humans, especially in the cerebellum, cortex, hippocampus and thalamus [210,211]. Interestingly, Trio expression decreases gradually in the adult brain [154,155,210]. Work done in *C. elegans*, *Drosophila*, rodents, as well as in human patients has reported that Trio plays a crucial role in the regulation of dendrite, dendritic spine, and synapse development and function [154,212,213,214]. Moreover, Trio was shown to be a crucial component of netrin-1/DCC-mediated axon outgrowth and guidance in *Drosophila* and rodents [35,81,84,85,215]. In addition, depletion of Trio in the mouse results in the malformation of hippocampal and forebrain structures, subsequently leading to defective learning ability [84,113,216]. In line with its extensive involvement in brain development, mutations in the *TRIO* gene have been associated with neurological and neurodevelopment disorders such as intellectual disability (ID), schizophrenia, developmental delay, and autism spectrum disorders (ASDs) [124,133,134,135,136,154,155,156,157,217]. De Ligt et al. [156] have identified two disparate missense mutations in the *TRIO* gene of two patients with severe ID. However, the study has not been able to link the *TRIO* gene to ID conclusively, as both patients have also harbored mutations in other well-established ID-related genes. A year later, a deletion mutation partially including *TRIO* gene has been reported to cause mild ID, developmental delay, behavioral defects, and facial dysmorphism [136]. Recently, more disease-causing *de novo* mutations have been identified in the *TRIO* gene. Ba and colleagues have identified four point mutations—three truncation and one frameshift mutation—leading to mild ID and behavioral defects [154]. Using rat hippocampal neurons, the authors have shown that the mutations leads to increased dendritic arborization and altered synaptic functions. In another study [157], several mutations in a region of *TRIO* gene encoding the GEF1 domain have been associated with ID and microcephaly. Finally, two recent studies [155,217], have reported the effect of disease-causing mutations of the *TRIO* gene in its encoded protein. They have concluded that the majority of the mutations are localized to the GEF1 domain of the protein, thereby reducing its Rac1-GEF activity. Collectively, these rodent and human case studies underlie the importance of TRIO, in particular its Rac1-GEF activity, in the developing brain.

#### 4.2.7. ARHGEF24 (Kalirin)

ARHGEF24, better known as Kalirin, is a Rac1-specific GEF protein [114,115,116]. Kalirin is expressed in a wide range of tissues such as brain, endocrine cells, liver, muscle, and heart. In neuronal tissues, Kalirin-9 and -12 are very abundant during development. These two isoforms localize to the growth cones and neurites of immature neurons. On the other hand, the adult brain expresses high level of Kalirin-7, which localizes to the postsynaptic compartments of excitatory synapses [117]. Kalirin has also been implicated in ephrinB-EphB-mediated dendritic spine morphogenesis [218]. Cahill et al. [114] have demonstrated that Kalirin-7-depleted mice show reduced cortical Rac1 activity and spine density, which lead to disease-related behavioral phenotypes such as impaired working memory, sociability, and prepulse inhibition problems. These findings are in line with the suggested function of Kalirin-7 in spine/synapse formation both in vitro and in vivo. Kalirin-7 has also been implicated in cocaine addiction in mice—its expression is upregulated in medium spiny neurons (MSNs) of the nucleus accumbens (NAc)—a brain area associated with drug addiction and reward pathways—upon chronic cocaine treatment. This upregulation leads to increased spine density, which is abolished in Kalirin-7-depleted mice [117]. Furthermore, reduced forebrain Kalirin-7 expression has been observed in schizophrenia and AD [115,163,164]. Several studies have also associated *Kalirin-7* gene with attention deficit hyperactivity disorder (ADHD), depression, epilepsy, and ischemic strokes [114,115,116,117]. Overall, similar to its homolog Trio, Kalirin seems to be of substantial importance in the CNS.

#### 4.2.8. ARHGEF36 (DNMBP)

ARHGEF36, better known as dynamin-binding protein (DNMBP), is a Cdc42-specific GEF protein [165]. DNMBP has high density at the periphery of presynaptic vesicle clusters, where it has been shown to colocalize with synapse-enriched proteins, amphiphysin-1 and dynamin-1 [165,166]. As a scaffolding protein, DNMBP brings dynamin and actin regulators together at the synaptic membranes to mediate events such as synaptic vesicle recycling. Because of its role in the recycling of amyloid precursor protein (APP), DNMBP is a promising candidate to be involved in AD pathology [165,166]. Therefore, two independent studies, targeting cohorts of late-onset AD patients in Japanese and Belgian populations, have associated SNPs in the *DNMBP* gene to late-onset AD [165,166]. Furthermore, it has been reported that the expression level of the *DNMBP* gene is reduced in AD brains by using postmortem brain sections from AD and age-matched unaffected individuals [165].

#### 4.2.9. ARHGEF42 (PLEKHG2)

ARHGEF42 is a Rac1 and Cdc42-specific GEF protein. It is only present in rodents and mammals [219]. Recently, a case study [219] has identified a missense mutation in *ARHGEF42* gene—p.R204W—that leads to reduced GEF activity and, consequently, impairment of Stromal Cell-derived Factor 1a (SDF1a)-stimulated actin polymerization. Five children affected with this mutation have shown profound MR, dystonia, postnatal microcephaly, and distinct neuroimaging patterns.

#### 4.2.10. ARHGEF44 (Puratrophin-1/PLEKHG4)

ARHGEF44, better known as PLEKHG4, is a GEF protein that activates RhoA, Rac1, and Cdc42. Its expression levels vary in different tissues—from high expression in the testis to the brain. In the mouse brain, PLEKHG4 is expressed in the brainstem, cerebellum, and midbrain [178,220]. However, only the cerebellar expression is sustained in the adult brain, especially in the Purkinje neurons. In line with the cerebellar expression of PLEKHG4, using positional cloning, Ishikawa and colleagues [178] have demonstrated that single nucleotide substitution in the 5′ untranslated region (UTR) of the *PLEKHG4* gene is associated with autosomal dominant cerebellar ataxia (ADCA), a group of heterogeneous neurodegenerative disorders.

#### 4.2.11. Alsin

Alsin is the product of *ALS2* gene. Alsin has GEF activity towards Rac, Ran, and Rab5, which makes it an important regulator of signaling through distinct small GTPases [171,172]. The *ALS2* gene produces two products by alternative splicing: the long (6394 nt) and the short (2651 nt) variants [171,172,173]. Mutations in the *ALS2* gene have been shown to cause infantile-onset ascending hereditary spastic paraplegia (IAHSP), juvenile-onset primary lateral sclerosis (JPLS), and amyotrophic lateral sclerosis (ALS). HSP, PLS, and ALS are genetically heterogeneous neurodegenerative diseases characterized by primary degeneration of motor neurons [171,172,173]. Initially it was proposed that the mutations that affect only the long variant cause JPLS, while the mutations that affect both variants cause the more severe form, ALS [171]. However, a study by Eymard-Pierre et al. [171] has argued against this by showing that a mutation affecting both variants of *alsin* transcripts shows no sign of lower motor involvement in affected patient.

#### 4.2.12. Faciogenital Dysplasia Protein 1 (FGD1)

FGD1 is a Cdc42-specific GEF protein [221,222]. Mutations in the different domains of *FGD1* gene have been implicated in the Aarskog–Scott syndrome, a rare X-linked recessive disorder that is characterized by short stature and exhibit unique skeletal and genital development [221]. In addition, a missense mutation in the *FGD1* gene was reported to be associated with XLMR and severe cognitive impairments [158].

#### 4.2.13. VAV1 and VAV3

The VAV family of proteins demonstrates GEF activity towards RhoA, RhoG, Rac1, and Cdc42. There are three VAV proteins in mammals: VAV1, 2, and 3. While VAV2 and 3 are expressed in several tissues, VAV1 is mainly restricted to haematopoietic lineage [223]. Using experimental autoimmune encephalomyelitis (EAE), a rat model of multiple sclerosis (MS)—a chronic inflammatory disease that damages myelin sheaths and nerve fibers—an association has been detected between VAV1 and MS [224]. The authors of the work have observed that VAV1 expression is elevated in rats and have suggested that VAV1 may have a role in MS. Furthermore, pursuing a preceding Japanese GWAS analysis of schizophrenia [125], Aleksic and colleagues have revealed VAV3 to be a candidate gene for schizophrenia based on Voxel-Based Morphometry and Mutation Screening [126].

## 5. Concluding Remarks

The Rho family of small GTPases and their regulators, in particular GAPs and GEFs, are crucial downstream components of guidance cue/receptor signaling pathways and dysregulation of their activity may lead to serious physiological complications. Consequently, more and more data are emerging which draw connections between the regulators of Rho GTPases and various diseases and disorders. Here, we presented an overview of the various GAPs and GEFs and their molecular implication in causing neurological disorders, especially in humans. However, our work does not tell the whole story as more findings related to the role of GAPs and GEFs in the CNS are being uncovered and even more have yet to be unveiled. A better understanding of these Rho GTPase regulatory mechanisms will certainly highlight novel targets for therapeutic interventions in the treatment of these neurological disorders.

## Figures and Tables

**Table 1:** GTPase-activating proteins (GAPs) and guanine nucleotide exchange factors (GEFs) in guidance cue/receptor signaling pathways.

Ligands	Receptors	GAPs	GEFs	References
Netrin-1	DCC	-	Trio DOCK180	[84,85,86]
Ephrin	Eph	α2-chimaerin	VAV2 and 3 Ephexin1	[67,87,88,89,90,91,92,93]
Semaphorin	Plexin/Neuropilin	p190RhoGAP	PDZ-RhoGEF LARG FARP2	[60,61,94,95]
Slit	Robo	vilse/CrGAP SrGAP	Sos	[63,64,96,97,98,99]

**Table 2:** GAPs and GEFs in neurological diseases and disorders.

Diseases and Disorders	GAPs	GEFs	Studied Organisms	References
Bipolar disorder	ARAP1, ARAP3, ARHGAP12, ARHGAP29, ARHGAP40, ARHGAP45		Human	[110]
Cognitive complications	ARHGAP15, 2-chimaerin	ARHGEF6, Trio, Kalirin	Mouse	[111,112,113,114,115,116,117]
Schizophrenia	ARHGAP18, ARHGAP33, Myosin IXb	Kalirin Trio VAV3	Human	[115,118,119,120,121,122,123,124,125,126]
Migraine	ARHGAP28		Human	[127]
Huntington’s disease	ARHGAP32	GEF-H1	Mouse, Rat	[128,129]
Autism spectrum disorders (ASD)	ARHGAP33, ARHGAP44	AKAP13, Collybistin, Trio	Human, Mouse	[121,122,130,131,132,133,134,135,136]
Depressive disorders	GMIP	Kalirin	Human, Rat, Mouse	[114,115,116,117,137]
Intellectual disability	OPHN1 srGAP3	GEF-H1, ARHGEF6, FGD1, Collybistin, Trio ARHGEF42	Human, Mouse	[131,136,138,139,140,141,142,143,144,145,146,147,148,149,150,151,152,153,154,155,156,157,158]
Seizure	RalBP1	Collybistin	Human, Mouse	[131,149,150,151,152,153,159,160]
Alzheimer’s disease	SH3BP1, α1-chimaerin	ARHGEF36, Kalirin	Human	[161,162,163,164,165,166]
Infantile epileptic encephalopathy	srGAP2		Human	[167]
Duane’s retraction syndrome	2-chimaerin		Human	[168,169,170]
Amyotrophic lateral sclerosis		Alsin	Human	[171,172,173]
Epilepsy	OPHN1	Collybistin, Kalirin	Human, Rat	[114,115,116,117,131,143,144,145,149,150,151,152,153]
Charcot–Marie–Tooth disease and polyneuropathy		ARHGEF10	Human, Dog	[174,175,176,177]
Attention deficit hyperactivity disorder		Kalirin	Human	[114,115,116,117]
Cocaine addiction		Kalirin	Mouse	[117]
Cerebellar ataxia		PLEKHG4	Human	[178]
